# Nucleic Acid Aptamers: Research Tools in Disease Diagnostics and Therapeutics

**DOI:** 10.1155/2014/540451

**Published:** 2014-06-22

**Authors:** Baby Santosh, Pramod K. Yadava

**Affiliations:** Applied Molecular Biology Laboratory, School of Life Sciences, Jawaharlal Nehru University, New Delhi 110067, India

## Abstract

Aptamers are short sequences of nucleic acid (DNA or RNA) or peptide molecules which adopt a conformation and bind cognate ligands with high affinity and specificity in a manner akin to antibody-antigen interactions. It has been globally acknowledged that aptamers promise a plethora of diagnostic and therapeutic applications. Although use of nucleic acid aptamers as targeted therapeutics or mediators of targeted drug delivery is a relatively new avenue of research, one aptamer-based drug “Macugen” is FDA approved and a series of aptamer-based drugs are in clinical pipelines. The present review discusses the aspects of design, unique properties, applications, and development of different aptamers to aid in cancer diagnosis, prevention, and/or treatment under defined conditions.

## 1. Introduction

Aptamers are relatively small biomolecules (typically oligonucleotides ranging from 40 to 180 nucleotides or peptides with 10 to 30 amino acid residues) whose three-dimensional structure confers on them the ability to bind their cognate ligands [1, 2]. The term “aptamer” is derived from a latin word “aptus” meaning “to fit” and introduced by Ellington and Szostak [1]. Nucleic acid aptamers can be chemically modified on the sugar backbone (i.e., 2′-fluro, 2′-O-methyl, phosphorothioate) to improve aptamer stability and functionality. Such nucleic acid modifications help in achieving optimal pharmacokinetic properties of selected aptamers towards chosen ligands. During the past three decades, aptamers have been generated against hundreds of molecular targets. Nucleic acid aptamers have been generated against various targets including organic dyes, metal ions, drugs, amino acids, cofactors, aminoglycosides and other antibiotics, base analogs, nucleotides, peptides, and numerous proteins of therapeutic interest like growth factors, enzymes, immunoglobulins, gene regulatory factors, and surface receptors [1–3]. Beside all these, aptamers are also selected against intact viral particles, pathogenic bacteria, and whole cancer cell as targets [3].

Nucleic acid aptamers selected from a library of random sequences by systematic evolution of ligands by exponential enrichment (SELEX) bind to the chosen ligands with high specificity and affinity [1, 2]. The SELEX process allows evolution or selection of molecules with highest affinity by their exponential enrichment among a population of random sequence nucleic acid library. It may be noted that SELEX is applicable in the case of nucleic acids due to the convenient intermittent amplification of affinity-selected molecules. During the SELEX process nucleic acid molecule can be amplified by RT-PCR or PCR. Some limitations of the use of antibodies can be overcome by the aptamers; for example, aptamers are generated* in vitro* and can be selected to target virtually any protein even toxins or nonimmunogenic proteins within a relatively short period of time, whereas antibody generation is limited by the need to use live animals [3]. In addition to this, aptamers are produced chemically in a readily scalable process and the selection process is not prone to viral or bacterial contamination [3]. Due to the smaller size of the aptamer, it may efficiently enter into biological compartment of the chosen target inside cells [4]. All these properties render aptamers superior for diagnostic application, offering greater sensitivity, reproducibility, and economy [4]. SELEX starts with a chemically synthesized random oligonucleotide combinatorial library of large sequence complexity, typically consisting of about 10^13^ to 10^15^ different variants of nucleic acid sequences, and involves the selection for oligonucleotides able to efficiently bind desired target molecules [4]. For the selection of RNA aptamers binding chosen target, the RNA library is obtained by* in vitro* transcription of a random DNA oligonucleotide library using T7 RNA polymerase before starting the first round of RNA SELEX process. Target binding function of nucleic acid aptamers is mainly dependent on their unique three-dimensional folding. The secondary structures of aptamers mainly consist of short helical arms and single stranded loops, defined by intramolecular base complementarity, whereas tertiary structures of aptamers result from a combination of these secondary structures with pseudoknotting of segmental sequence complementarity of loops and bulges and allow aptamers to bind target by noncovalent interactions like Van der-Waals interactions, hydrogen bonding, topological compatibility, stacking of aromatic rings, and electrostatic interactions [5].

## 2. Designing Aptamer Library and Basic Principle Underlying SELEX 

SELEX is started with a population of different random sequences flanked by defined sequences. The defined sequences are placed to ensure amplification of all different sequences in the selected population by polymerase chain reaction (PCR). The primers designed should anneal specifically to the template without forming “primer dimer” or secondary structures. Typically, up to 20-nucleotide long primers are used for PCR and can be synthesized with good yield. For selection of RNA aptamers, T7 RNA polymerase promoter sequence is required 5′ to the PCR template sequence within the primer design (Figure 2). In principle, aptamer libraries up to 10^20^ oligonuclotides are technically feasible but are rarely used in practice [6]. The major considerations made while designing libraries are summarized below.

### 2.1. Type of Randomization

NA aptamer randomization depends upon sequence information of aptamer random sequence region. Three types of randomization are employed in designing aptamer random sequence region, that is, partial, segmental, and complete. Partially randomized (doped) library is used for selecting new functional molecules by taking in consideration the motifs whose structure and fuctions are known to bind with the target molecule [7]. Knowledge of structural and functional motifs suggests and suffices for the use of partial random sequences in random sequence region. The extent of randomization and coverage of variant sequences flanking the motif are the basis of designing and synthesis of the partial randomized pool. It corresponds to mutation at constant rate for each nucleotide in sequence, resulting in an array of point mutations similar to nature but at much higher frequencies [7]. In segmental randomization, a compromise between partial and complete randomization is made in longer oligonucleotide sequences. Completely random library design allows launching aptamer selection with minimal or no prior knowledge of structure of either the target or the aptamer [7].

### 2.2. Length of Random Sequence Region (An Aptamer Unit)

**Figure 1:**
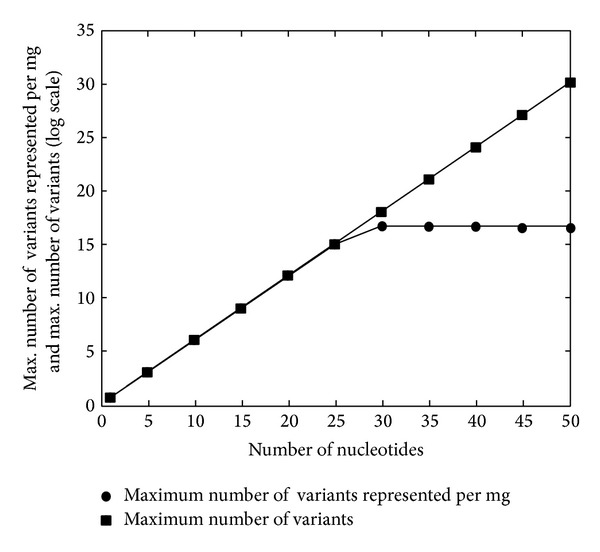
Maximum number of variants (filled squares) and number of variants per milligram (solid circles) of oligonucleotides as a function of the number of nucleotides in the nucleic acid sequence in aptamer library.

It is very important to choose the length of the random region while selecting for an aptamer. It is a wishful assumption that libraries with a longer random region may facilitate selection of more efficient aptamers because longer sequences are able to form a wider range of different three-dimensional structures [7]. The longer sequences offer a large number of short structural motifs in addition to diverse and large motifs [8]. If the length of this region is *N* nucleotides long, it will give a maximum of 4^*N*^ possible sequence variants which means a theoretical maximum of 4^*N*^ probable conformations. Short, randomized, nucleic acid segments of 20 to 50 nucleotides provide a vast sequence space for novel nucleic acid structures and functions [8]. If a target has intrinsic nonspecific and weak affinity for nucleic acids, then a partial or segmental random sequence pool of 30 to 60 nucleotides based on natural ligands will yield aptamers. Since the total mass of oligonucleotides used for starting SELEX is limited, it puts a natural as well as a practical limit on the total number of variants that can be taken, irrespective of the size of the oligonucleotides and theoretical maximum number of variants it may potentially form. Therefore, it is statistically impossible and practically nonfeasible (presented in Figure 1) to achieve the synthesis of a library with the maximum theoretical diversity. For the practical reasons,* in vitro* selections of nucleic acids are typically limited to 4^30^ (approx. 10^20^) different sequences.

### 2.3. Chemistry of Aptamer Library

Chemistry of the oligonucleotide pool also affects the selection method to be employed for the development of aptamers with affinity for chosen ligands. With increasing size of the DNA molecule, a larger mass would be needed to represent each possible variant in the pool. As the number of nucleotides increases, that is, the length of random nucleotide sequence, maximum number of variants per unit mass (mg) shows saturation and in fact declines for larger aptamers (Figure 1). So larger amounts of DNA are required for including maximum possible number of structural variants.* In vitro* selection can also make use of modified nucleotides (NTPs) which further confers the desired pharmacokinetic properties on the aptamers. The modified nucleotides can affect the chemistry, which in turn can bias the course and outcome of SELEX [7]. The T7 RNA polymerase can incorporate many modified ribonucleotides such as 2′-flouro and 2′-amino rNTPs or phosphorothioate nucleotides [6, 7].

**Figure 2:**
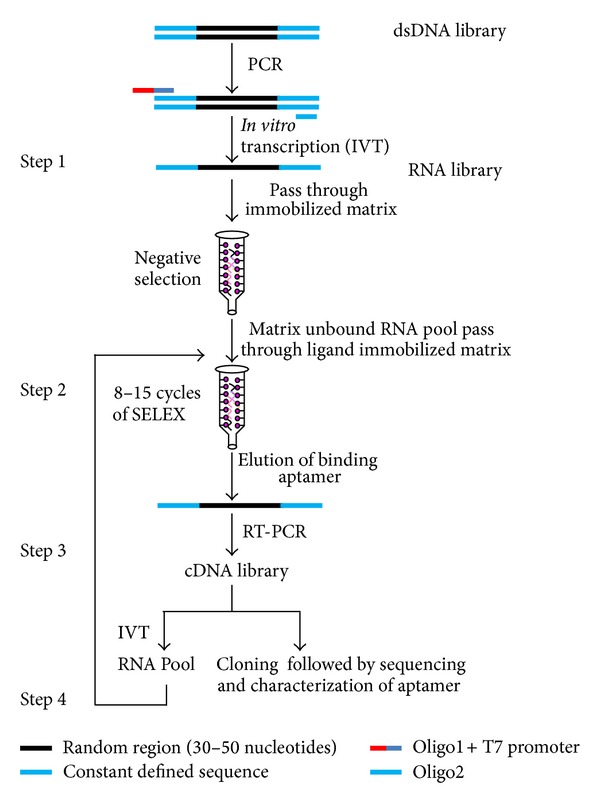
Schematic representation of SELEX technology. A suspension of ligand coated matrix is often used instead of column.

The basic approach behind the SELEX protocol is to minimize the total library pool size towards relatively small number of binding partners. This also includes enrichment of high affinity and high specificity binding motifs by repetitive cycles of affinity selection and enzymatic amplification simultaneously. This process is mainly divided in four steps, namely,* in vitro* transcription, affinity binding and elution, and reverse transcription (Figure 2) [1, 2], as follows:

1. synthesis of library of random oligonucleotides flanked by defined oligonucleotide sequences and incubation with cognate ligands after negative selection,
2. binding the cognate aptamers and washing off the nonbinding molecules from random oligonucleotide sequences,
3. amplification of selected aptamers by RT-PCR (RNA aptamers) or PCR (DNA aptamers),
4. cloning and characterization of selected aptamers and further enrichment of high affinity aptamers by iterative rounds of selection.

Negative selection for subtraction is a crucial step to minimize enrichment of nonspecifically bound oligonucleotides with the matrix absent of ligand [3, 4]. For this reason, the random pool of RNA library is passed through an affinity matrix/beads without immobilizing ligand. Further, beads are washed with appropriate buffer and unbound RNA pools are collected and the above process is repeated with ligand immobilized affinity matrix/beads. Next, binding aptamers are eluted with competitor in elution buffer after washing and finally amplified by PCR or RT-PCR. The sequences so selected are used as template for the next round of RT-PCR or PCR. The same cycle is repeated 10 to 12 or more times depending on nature and concentration of ligand, design of the starting random DNA oligonucleotide library, selection condition, ratio of ligand molecules to oligonucleotide, or the efficiency of the partitioning method to get an enriched pool of RNA molecules which specifically bind the desired ligand with high affinity [9]. RNA aptamers have been selected against numerous targets listed in Table 1.

## 3. Salient Features of Therapeutic Aptamers

Aptamers are increasingly being used as a replacement option for antibody mainly because of the special features described in the following sections.

### 3.1. High Specificity and Efficiency

The method for nucleic acid aptamer selection is quite simple and yields highly specific aptamers within a short time [4, 6]. It has been shown that the aptamer often possesses high affinity towards the selected molecule as compared to antibodies and often yields Kd value in the nanomolar range [4, 5].

### 3.2. Nonimmunogenic, Nontoxic, and Nonrecalcitrant Nature

There are no reports demonstrating toxicity or immunogenicity of aptamers. It is observed by clinical, cellular, or biochemical measures that high doses of aptamers (10 mg/kg daily for 90 days) are not toxic in rats or woodchucks [44]. While the efficacy of many monoclonal antibodies can be severely limited by immune responses against antibodies themselves, it is quite difficult to raise antibody response against aptamers (most likely because aptamers cannot be presented by immune cells (T cells) via the major histocompatibility complex and the immune response is generally trained not to recognize nucleic acid fragments as nonself entities [45].

### 3.3. Route of Administration

Nucleic acid aptamers can be administered by either intravenous or subcutaneous injection, whereas most antibody therapeutics are administered via intravenous infusion. Due to low solubility, large volumes are necessary for therapeutic monoclonal antibodies. With good solubility (>150 mg/mL) and comparatively low molecular weight (aptamer: 10–50 kDa; antibody: 150 kDa), aptamers are advantageous for tumor penetration and blood clearance. Aptamer bioavailability via subcutaneous administration is >80% in monkeys [46]. Therefore, for the systemic delivery of nucleic acid based therapeutics, subcutaneous administration has been suggested as an effective strategy [46].

### 3.4. Optimal Pharmacokinetics and Clearance from the Living System

For improving the stability of aptamers, pyrimidine as well as purine nucleotides can be modified at their 2′ positions and tested for their ability to accommodate stabilizing modifications like 2′-O-methyl groups [47]. These modified nucleotides can be introduced either chemically or enzymatically. Protection against exonucleases can be provided by modifications at the 5′ caps (such as PEG adducts) and 3′ ends (e.g., by addition of a 3′-3′-linked thymidine cap) to increase aptamer retention time in the blood [47]. Such modifications have a profound effect on aptamer retention in animals, extending aptamer half-life from minutes (no PEG) to several hours (40 kD PEG) [45]. Conjugation with 40 kD PEG at its 5′ terminus increases the* in vivo* half-life of the thrombin aptamer from 24 min to 6 h in rats, with little or no effect on thrombin binding affinity [48]. However, these stable forms also are eventually cleared from the system [48].

### 3.5. Possibility of Economic Scale-Up and Stability

Therapeutic aptamers are chemically synthesized and consequently can be readily scaled as needed to meet production demand. Therapeutic aptamers are chemically robust. They are intrinsically adapted to regain activity following exposure to heat, denaturants, and so forth and can be stored for extended periods (more than 1 year) at room temperature as lyophilized powders [45].

## 4. Application of Aptamers 

**Figure 3:**
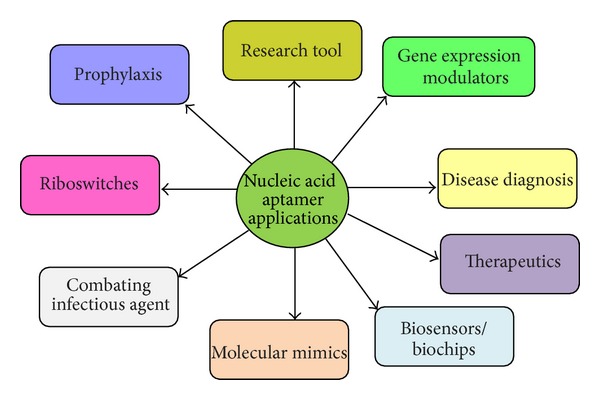
A summary showing potential application of nucleic acid aptamers.

The above-mentioned properties of aptamer specificity toward target ligands make them an ideal tool for diagnostics and therapeutics. Aptamers have been extensively used in analytical applications. Nucleic acid aptamers have wide applicability as exemplified in the following sections (Figure 3).

### 4.1. Aptamers in Prophylaxis

The applicability of aptamer is not limited to targeted delivery to their cognate molecules, but they can function for prophylactic help as well. These molecules could either be intracellular (e.g., beta-catenin, thyroid transcription factor, etc.), extracellular (e.g., VEGF, Tenascin-C, or small molecules such as ATP, AMP, and theophylline), or cell-surface targets (e.g., PSMA, Mucin-1, gp120, EGFR, etc.). Nucleic acid aptamers have been projected as convenient molecular mimics of antibody to provide the basis for developing prophylactic idiotypes in much the same way as monoclonal antibodies have been used. Nowadays, increased chances of catheter associated urinary tract infections (CAUTIs) are imposing a big challenge [49].* Proteus mirabilis *(causative agent of CAUTIs) blocks the flexible tube connecting body cavity, which disallows fluids to pass into or out of it by forming a crystalline biofilm [49]. Blockage is often not detected until major complications arise in the patients such as pyelonephritis, septicaemia, and shock. Early diagnosis and prophylactic aid are needed to deal with many such diseases. Therefore, high affinity DNA aptamers (PmA102, PmA109) for the improvement against* Proteus mirabilis* were selected using cell-SELEX in combination with* in silico* maturation (ISM). These aptamers would be utilized for biosensor development to get an insight into pathogen associated membrane proteins [50]. In another example of prophylactic aptamer, CD28 aptamers (CD28Apt7-dimer, CD28Apt2) were also developed that boost antitumor immune response induced by idiotypic vaccination as well as by blocking B7-CD28 interaction by binding to it [51]. TCR-mediated signaling and engagements of CD28 are basically required for efficient induction of the T-cell response. This costimulatory CD28 molecule plays important roles in induction and maintenance of antiviral T-cell responses. The efficacy of promoting strong cellular and humoral response by CD28Apt7-dimer is key to promising approach in cancer immunotherapy. The same aptamer works as an inhibitor for CD28 functions and, after dimerization, it functions as a potent adjuvant [51].

### 4.2. Aptamers as Riboswitches

Riboswitches are naturally occurring RNA sequences with specific structural recognition of cellular metabolites to modulate gene expression. The untranslated regions of many mRNAs are known to act as sensors of metabolic pool and reorganize themselves resulting in altered efficacy of translation [52]. This helps the cells switch their expression platforms. Such noncoding RNA sequences are naturally occurring aptamers and referred to as riboswitches [52]. These are* cis*-acting genetic control elements that regulate metabolic genes via structured sequences present in 5′-untranslated region of mRNA. Binding of metabolites to aptamer domain is independent of protein factors [52]. Discovery of riboswitches illustrates biological relevance of molecular recognition by natural RNA aptamers [53]. It has been speculated that we should find new aptamers in hitherto unexplored regions of the genomes [54]. These structured RNA domains are widespread in bacteria and help in modulation of many fundamental biochemical pathways by sensing the metabolites such as adenine, guanine, FMN, glycine, and lysine [52]. Guanine sensing riboswitches are quite similar to adenine riboswitch; binding of adenine promotes mRNA transcription by preventing formation of a terminator stem [53]. Both belong to the same class of riboswitch, that is, purine riboswitch [55]. A riboswitch has been reported in plants and fungi that bind to TPP (TPP riboswitch) [53]. Generally, riboswitches have two domains, an aptamer domain that binds the effector ligand and an expression platform that transduces the binding event into a change in gene expression [55]. Riboswitches are also involved in a form of feedback inhibition via binding of metabolite, further decreasing the production of related gene products and resulting in transcriptional attenuation by preventing formation of full length mRNA or inhibition of translation initiation.

### 4.3. Aptamers in Disease Diagnosis

**Figure 4:**
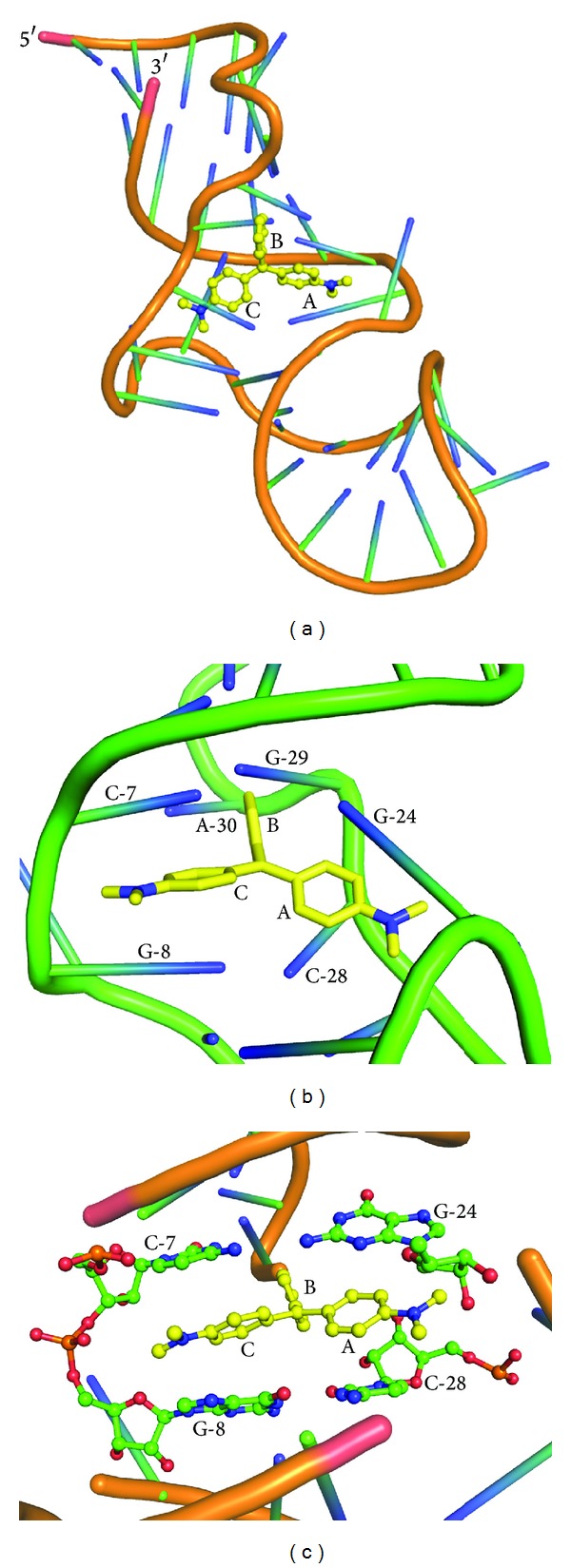
Solution structure (NMR) of malachite green RNA aptamer: (a) shows malachite green ligand bound to RNA aptamer backbone, (b) shows residues involved in malachite stacking, and (c) shows ball and stick model of RNA residues involved in malachite stacking interaction (yellow colour ring structure represents malachite green as ligand), adapted from Flinders et al. [59].

Clinical diagnosis of disease with the help of aptamers is attractive due to their small size, stable folding structure, and economy. There has been rapid advancement in application of such aptamers in disease diagnosis, imaging, and new biomarker discovery. Aptamers can detect very low amounts of diseased or tumor cells for which they are selected. For example, immobilized anti-EGFR RNA aptamer on a chemically modified glass surface can determine the presence and/or extent of GBM (glioblastoma) tumor cells [56]. EGFR is the most common oncogene in glioblastoma [56]. It is barely detectable with a small number of diseased cells. By using this novel approach, it has become possible to make early diagnosis and monitor residual diseased cells after surgical removal of tumor [56]. Aptamers can also be used in lieu of antibodies in flow cytometry to detect a wide variety of cells such as human cancer cells. For example, newly developed CD30 aptamer probe acts as an antibody free replacement option for the diagnosis of CD30 expressing lymphomas [57]. Further development is needed to push this technology to make it preferred option in the clinic. The future of the use of aptamer-based probe in noninvasive imaging of many potential clinical applications, such as lesion detection and monitoring of treatment, is becoming increasingly promising and needs further validation studies to benefit patients. The principle of aptamer-based probe designing is similar to that of developing aptasensor. Such aptamer-based probe usually consists of two domains: one is sensing domain which recognizes the target molecule and the other is signaling domain which gives signal through a reporter molecule. The reporter molecule can be fluorescent or radionuclide. An aptamer-based diagnostic approach was developed to detect malachite green (MG), a suspected carcinogen, in fish food. This MGRNA aptamer is used as alternative recognition tool over conventional antibody (Figure 4) [58]. Leucomalachite green (LMG) is a primary metabolite of MG and persists longer in fish tissues. However, this MG aptamer shows negligible affinity towards LMG and is specific for MG. Hence, the detection of malachite green by using this RNA aptamer is preceded by an oxidation step that ensures conversion of all LMG to MG [58].

Biomarkers are important and crucial in diagnosis and treatment of cancer [60]. They can be expressed in different forms such as proteins unique to cancer type and subtype. It could be soluble (secretory) or membrane attached. Importantly, cell-SELEX for novel aptamer selection is being exploited for biomarker discovery without prior knowledge of the cell markers [60]. Protein tyrosine kinases (PTK7) like molecules were found to be highly expressed in a series of leukaemia cell lines by using whole cell-SELEX in a two-step process, that is, aptamer selection followed by biomarker discovery [61]. Emerging application of aptamers includes detection of protein biomarker at picomolar concentration and in biomarker discovery. For example, SPRI (surface plasmon resonance imaging) methodology can sense human thrombin at a concentration of 500 fM and VEGF protein at biologically relevant concentration of 1 pM in biological samples by forming a sandwich structure (RNA aptamer-protein biomarker-antibody-HRP) [62]. This technique is utilized for finding protein biomarkers at very low concentrations [62]. Formation of surface aptamer-protein-antibody complex is detected by adsorption of proteins on to immobilized RNA aptamer microarrays. After binding of protein, SPRI response signal is further amplified by HRP conjugated antibodies by forming localized precipitation/colour reactions [62]. By using SPR imaging assay, Li et al. first immobilized an array of three thrombin binding RNA aptamers to enrich serum thrombin followed by signal amplification by horseradish peroxidise conjugated antibody against human thrombin [62]. It is recommended and required for prediagnosis of disease and tracking followup procedure during therapeutic application. In addition to this, it is a powerful method to keep rapid detection and profiling of protein biomarkers in blood or serum. This method is dependent on the use of two different binding sites on target by both RNA aptamer and an HRP conjugated antibody. In the future, aptamers would possibly eliminate the use of antibodies from the detection approach by replacement with second-generation biotinylated nucleic acids or peptide aptamers.

### 4.4. Combating Infectious Disease

Aptamer technology offers feasible alternative options for addressing the challenge of early diagnosis in human health [63]. A single-stranded DNA aptamer was developed against* botulinum* toxin [64]. This DNA aptamer was selected against aldehyde inactivated toxins and a short peptide fragment of 1177 to 1195 aa of the heavy chain in a single microbead based selection assay. The DNA aptamer exhibits affinity with toxoid in nanomolar range (Kd: 3 nM). Further progress in this field was marked by the selection of DNA aptamers against cholera toxin and* Staphylococcal enterotoxin* B (SEB) [65]. The cytotoxin ricin is derived from castor bean plant that disrupts translation by depurinating a conserved site in eukaryotic rRNA [63]. RNA aptamer was developed against the Ricin A chain with Kd 7.3 nM [66]. Selected RNA aptamers that recognize and potentially inhibit ricin might be used as prophylactic or therapeutic agent or function as a biosensor for the detection of aerosolized ricin contaminated by the toxin [66]. DNA aptamer (SSRA1) against ricin B chain can also serve as a diagnostic and preanalytical tool for direct ricin detection [67]. Biotoxins from pathogenic bacterial strains in humans are solely responsible for severe health problems that demand innovative technologies with greater sensitivity and specificities [63]. Although, traditional antibody based methods are quite sensitive in some cases, the demand for robust aptamer technology is quite high. RNase resistant RNA aptamer was selected against OmpC protein of* Salmonella typhimurium* with high affinity (Kd: 20 nM) and specificity [36]. It did not show binding with any other Gram-negative or Gram-positive bacteria. Thus, this RNA aptamer can diagnose food borne sickness caused by* Salmonella typhimurium*. These may find use in prophylaxis or targeted delivery vehicles.

### 4.5. Aptamers as Biosensors/Chips

Aptamers are also used in biosensors due to the behaviour of ligand induced conformational changes. Such aptamers work as molecular beacons and may be used for the detection of environmental contaminant and monitoring carcinogen or to check the level of drugs in the blood [3]. Over the past decade, the dependence and applicability of aptamers have steadily increased. Aptamers are used as fascinating tools for biosensor and biosecurity applications. In the novel approach, aptamer magnetic bead electrochemiluminescence (AM-ECL) sandwich assay was used for the selection and development of ssDNA aptamers against autoclaved anthrax spores [68]. This cost effective, ssDNA aptamer acts as* in vitro*-generated receptor for use as a biosensor for molecules used in biological warfare. Another sensory system (RNA aptamer) for opium alkaloid codeine, a class of benzylisoquinoline alkaloids (BIAs) and metabolite in opium alkaloid biosynthesis pathway, can precisely measure the codeine concentration. Two best codeine binding RNA aptamers (FC5 and FC45), with Kd 2.5 and 4.0 *μ*M, respectively, are good enough to discriminate codeine from its structural analog [69]. Another example of aptamer-based sensor is utilized in detection of cocaine at concentration of 1–100 *μ*M in human blood serum by a “split aptamer” [70]. The split aptamer is utilized as recognition element for sensing small molecules, that is, cocaine, metal ions, and adenosine [71]. Designing of such aptamer-based strategies involves the engineering of an aptamer into two fragments of ssDNA and interaction with target causes ligand induced conformational change, resulting in assembling of both nucleic acid fragments by forming junction trimer complex [70].

Aptamers can be used as chip based biosensor array by immobilizing fluorescent labelled nucleic acid aptamer on a glass slide where they can still rotate at a rate that corresponds to their apparent volume and mass [72]. The binding implies a change in the mass and consequently in the rotation rate which conditions the fluorescence polarisation. This technique has been proven to be efficient even with complex biological matrices such as human serum or cell extracts [72]. This aptamer biosensor array provides an easy detection of multiplex analyte in complex biological mixtures.

### 4.6. Aptamers as Modulators of Gene Expression

Chimeric RNA molecules have been constructed by fusion of aptamers with targeted ribozyme [73]. Such chimeric constructs act as ligand responsive “aptazymes.” Aptazymes can serve as extremely sensitive molecular sensors in which the aptamer domain recognizes the ligand and the catalytic domain cleaves and splices the target. These are engineered nucleic acid whose activity can be regulated by small molecules or cofactors. A group 1 aptazyme (allosteric enzyme) engineered by inserting thymidylate synthase intron in tandem with theophylline aptamer has been projected as a genetic regulatory switch in gene therapy [73]. This aptazyme shows dependence over theophylline both* in vitro* and* in vivo* for its activity of ligand dependent splicing. Insertion of aptamers into the 5′-untranslated region of mRNA provides a handle for translational control of expression of specific genes in living cells. Translation of such aptamer RNA constructs can be regulated by reversible ligand dependent conformational change of aptamer domain [74].

Cancer cells show altered expression of signaling pathways due to mutation in one or more than one gene. There are upregulation of oncogenes and downregulation of tumor suppressor genes. Cancer cells exhibit tendency for uncontrolled cell division and successfully escape from conventional apoptosis pathways. The Wnt (beta-catenin) signaling plays an important role in controlling human tumorigenesis. Beta-catenin serves as an effective anticancer drug target.* In vitro* selected TCF RNA aptamer binds with T-cell factor and inhibits the tumorigenic function of beta-catenin in colon cancer cells by modulating beta-catenin target genes such as cyclin D1 and matrix metalloproteinase-7 [75]. CD28 is constitutively expressed on naive T lymphocytes and acts as a potent costimulatory signal for T-cell activation. After signaling from CD28 in conjunction with other T-cell surface molecules, T cells produce and secrete interleukins. Expression of B7, the ligand for CD28, is upregulated on the surface of antigen presenting cells (APCs) after getting signals from ligand of Toll-like receptor. This costimulatory signal is very important for the production of interleukins; lack of this signal causes stage of anergy, that is, impaired response to its antigen. CD28 aptamers modulate immune response as agonist and also as antagonist. This aptamer acts as an antagonist by blocking the interaction of B7 with CD28 and functions as an agonist via elevating vaccine induced immune responses in cancer immunotherapy [51]. This aptamer-vaccine combination has shown preliminary success in mice [51].

### 4.7. Aptamers as Therapeutics

Due to their merits as cognate molecules, aptamers are quite attractive for therapeutic applications too. As therapeutic agent, RNA aptamers have advantages over other classes of RNA tool like short hairpin RNA (shRNA), small interfering RNA (siRNA), ribozyme, or antisense oligonucleotides (AS OGNs), [76] in terms of intracellular targeting capabilities, direct binding to extracellular targets, and sequestering/inhibiting their function. Aptamers can be improved by labeling or conjugation with siRNA or toxins in therapeutic applications [77, 78]. It has also been found to be a powerful tool for delivery of variety of therapeutic agents such as small molecules, peptides, or toxins for the treatment of human diseases [79]. Currently a number of such aptamers are available in clinical and preclinical trials for treatment of cancer and other human diseases (Tables 1 and 2).

The inhibition of viral invasion and replication using nucleic acid aptamer is another important area of research. An RNA aptamer “Macugen” is targeted against the angiogenic cytokine VEGF and its binding prevents choroidal neovascularization. In 2004, US Food and Drug Administration (FDA) approved the first aptamer-based drug Macugen or Pegaptanib sodium (aptamer against VEGF, Eyetech Pharmaceutics/Pfizer) [80]. This was a milestone in the application of aptamer technology. The VEGF protein has a central role in physiological and pathological angiogenesis through binding to its receptors (VEGFR1 and VEGFR2) that activate downstream signaling. The VEGF gene expresses four major isoforms of 121, 165, 189, and 206 aa proteins through alternative splicing. Out of the four, VEGF_165_ is the dominant isoform, primarily responsible for pathological neovascularization in age-related macular degeneration (AMD) and diabetic macular edema (DME) [80]. Researchers have selected RNA aptamers against this VEGF_165_ selectively, leaving other isoforms unaffected and modified them to increase their stability in serum by fluorination, methylation, and addition of a 5′-poly ethylene glycol moiety [29, 80]. This RNA aptamer showed no toxicity and has very long half-life in pharmacokinetic studies and was developed as a drug approved for treatment of AMD and DME [80].

Similarly, EGFR protein family consists of four closely related receptor tyrosine kinases, namely, EGFR or ErbB-1 and its three homologs ErbB-2 or HER2/neu, ErbB-3 or HER3, and ErbB-4 or HER4 that can form homo- and heterodimers after receiving signals from EGF, TGF-*β*, and heregulin ligands to activate downstream signaling cascades [19]. Mutation in expression or activity of EGFR family protein leads to a wide variety of cancers such as glioblastoma and breast, ovarian, stomach, bladder, salivary gland, and lung cancer. It has been shown that EGFR binding aptamers (E07) could be a promising candidate for cancer therapy by blocking receptor activation and inhibiting cancer cell progression [19]. Further, DNA aptamer (HB5) against HER2 was isolated and characterized for its target binding [35]. HER2 and HER3 (homologs of EGFR family) both share homology between the extracellular domains (ECD). In spite of this, A30 (RNA aptamer) shows no binding to the ECD of HER2 even at concentrations far above those used in inhibition studies [90]. A30 RNA aptamer is specific and shows high affinity for HER3ECD and inhibits the heregulin-induced activation of HER3 receptor following inhibition of growth of MCF7 cells [90]. Further, functional validation is still needed to confirm the antitumorigenic properties of the above-mentioned nucleic acid aptamers.

Prostate specific membrane antigen (PSMA) is a unique marker for prostate cancer cells. PSMA specific RNA aptamers (xPSM-A9 and xPSM-A10) were isolated and used as intracellular delivery tools in conjugation with siRNAs or shRNAs for specifically targeting cancer cells instead of normal cells [91]. In this aptamer siRNA chimera, RNA aptamer (A9, A10) when conjugated with different siRNAs such as lamin A/C, polio-like kinase 1 (PLK1), cancer survival gene, and BCL2 gene caused more pronounced regression of PSMA specific tumors* in vivo* [24, 92]. Radiotherapies as well as chemotherapies are powerful conventional tools for cancer therapy, but the disadvantage of these methods is their nonspecificity for target cells as it kills normal cells as well. However, there are DNA repair genes and signaling cascades that play role in overcoming this disaster through DNA repair mechanisms up to a certain level; beyond this threshold cells undergo apoptosis. RNA aptamer shRNA chimera (A10-DNA-PK) has been used to target such vital genes as DNA activated protein kinase to increase the radiosensitivity of prostate cancer cells [93]. This approach allows low level of ionizing radiation to inactivate cancer cells and decreases the necessity of exposure to surrounding tissues such as the bladder.

The surface glycoprotein gp120 of the human immunodeficiency virus interacts with host CD4 receptors leading to initiation of membrane fusion and delivery of viral RNA and enzymes to host cells. Aptamer siRNA chimeras have been developed to silence virus replication and propagation in T cells. In this chimera, siRNA is used against the HIV-1 tat/rev region [16]. In combination with pRNA, a dual functional RNA nanoparticle was developed for targeted inhibition of HIV [94]. Modified pRNA-aptamer-siRNA chimeras can be functionally processed by dicer to silence target gene expression and provide a novel tool for cancer therapeutics.

### 4.8. Aptamers as Research Tools

Aptamers have wide applicability in pathway elucidation through their effect as specific inhibitors of intracellular signaling pathways. One example of such an inhibitory aptamer is the mitogen activated protein kinase (MAPK) RNA aptamer [95]. MAPK pathway is involved in various physiological responses such as cell proliferation, apoptosis, and differentiation. Mutations in MAPK lead to inappropriate activation of the MAPK signaling cascade and ultimately promote diseased states such as cancer in humans [95]. Inhibitory RNA aptamers have been used as novel anticancerous therapeutic tools either alone or in conjunction with siRNA as mentioned in Table 1. [16, 24]. Applicability of aptamers is extended to studies in molecular biology and immunology as targeted modulator of various functions [57]. The advancing aptamer technology holds promise to develop novel therapeutic molecules which may soon find their own way to routine laboratory and clinical procedures.

### 4.9. Aptamers as Molecular Mimics

*In vitro* selected nucleic acid aptamers serve as molecular mimics as well. Those aptamers can be selected employing simple chemistry of selection. Pathogen induced immune defense involves initiation of recruitment of inflammatory cells (leukocytes and platelets) to target sites (endothelial cells) [96]. In such processes, cell-adhesion molecules are involved in three types of cell-cell interactions (circulatory inflammatory cells to endothelium, circulatory inflammatory cells to arrested inflammatory cells, and among circulatory inflammatory cells) [96]. P- or L-selectin binding nucleic acid aptamers on mesenchymal stem cells (MSCs) are selected and bind to protein receptors on adjacent cells [97]. Such aptamers resemble artificial “adhesion ligands” [97]. Further, chemically engineered MSCs modulate cellular functions via cell-cell interaction. To expand the utility of aptamers, composite RNA aptamers (aptabody) were also designed and selected to function as antibodies in immunological assays [98]. Another example of aptamers as molecular mimics is a Spinach aptamer based on RNA mimicking green fluorescent protein [99]. Certain RNA sequences complexed with flurophore mimic GFP in living cells [99]. Generally, proteins are fused with reporter molecule such as green fluorescent protein or its derivatives to measures the expression* in vivo *or inside the living cells. Similarly, Spinach aptamer fluoresces inside living cells in presence of provided fluorophore molecule, that is, 3, 5-difluoro-4-hydroxybenzylidene imidazolinone (DIHBI) [99]. A report of mRFP1-Spinach construct showed* in vivo* transcript measurement and protein synthesis from the same mRNA simultaneously. Spinach aptamer sequences can serve as good tools to characterize transcription and translation inside cells. Such aptamer requires short RNA sequences with provided fluorophore [99].

## 5. Conclusion and Future Prospects

The importance of the applicability of aptamers has significantly increased with recent advancement in nanotechnology, microfluidics, microarray, and other technologies for application in the clinical field. Aptamers have been proven as multifunctional molecules having tremendous paramedical and medical applications. A number of such aptamers are in various stages of clinical trials. Recent advancement in aptamers selection and development can potentially deal with targeted drug delivery, targeted inhibition of protein function, and regulation of gene expression. Aptamers can also help in early cancer detection and alleviate detection complications. Aptamers compete with antibodies and offer promising tools in different applications. Targeted delivery, target specificity, and high affinity sum up into a higher application index in favor of aptamers, although, some limitations also exist with aptamers tools such as the stability of aptamers with modified nucleotides, intake, and its retention time inside the living system. Despite the enormous possibilities, nucleic acid aptamers suffer from certain limitations. These include a typical molecular mass being in excess of 10 kD, relative unstability of unmodified RNA aptamers, and possible off-target effects. However, further studies can confirm and validate outcomes of this selective approach by profiling the targeting specificity of the chosen ligand. Aptamers binding with transition state analogues of biochemical reaction may be expected to catalyze the respective biochemical reactions. An aptamer called “Spinach” mimics green fluorescent protein and promises use as tool for characterization of gene expression. This could be used in other applications such as RNA imaging. Variants of such aptamers offer great potential applications in synthetic biology.

## Figures and Tables

**Table 1:** A list of aptamers generated against various ligands.

Nature	Aptamer name	Ligand/target	Kd (nM)	Disease	Reference
	RNA aptamer	RIG1 (1-925aa)	≤37.2	Antiviral activity via modulating IFN*α*/*β* production	[10]
	AFP aptamer	AFP (fetal protein)	33	Hepatocellular carcinoma	[11]
	Class III GSH aptamer 8.17	Glutathione	41.8	Breast cancer (human breast adenocarcinoma cell line, MCF-7)	[12]
	MDA aptamer M1	MDA (4,4′-methylenedianiline)	450	Carcinogenic and DNA damaging agent	[13]
	RNA aptamer	Rev peptide	19–36	HIV	[14]
	RNA aptamer S66A-C6, RNA aptamer S69A-C15	V3 loop of gp120, HIV-1RT	406, 637	HIV	[15]
	Anti-gp120 aptamer chimera	Glycoprotein 120 (gp120)	na	HIV	[16]
	Class I and Class II AChR aptamer	Acetylcholine receptor (AChR)	2 and 12, respectively	Neuromuscular disorder	[17]
	RNAapt TH14	Beta-Secretase BACE1 (B1-CT)	280	Alzheimer's disease	[18]
	Anti-EGFR aptamer (E07)	Epidermal growth factor receptor	2.4	Breast and lung cancer	[19]
RNA	DBL1*α*-specific RNA aptamer	Erythrocyte membrane protein 1 (PfEMP1)	33 inhibitory conc.	Malaria	[20]
	2′F RNA and 2′NH2 aptamer	Human keratinocyte growth factor	0.0003–0.003 and 0.4	Cancer	[21]
	RNA aptamer (10th rounds 4°C)	L-selectin	17	Inflammation	[22]
	RNA aptamer	NF-kB p50 homodimer	5.4 ± 2.2	Cancer and inflammation	[23]
	Anti-PSMA chimera	Prostate specific membrane antigen	na	Prostate cancer	[24]
	Aptamer number 5	T-cell factor 1	100	Colon cancer	[25]
	TTA1	Tenascin-C	5	Glioblastoma, breast cancer	[26, 27]
	RNA aptamer	TGF*β* type III receptor	1	Ovarian cancer	[28]
	Pegaptanib sodium, NX1838	VEGF 165 (vascular endothelial growth factor 165)	0.05	Age related macular degeneration	[29]
	RNA aptamer 22	WT1	700	Wilm's tumor	[30]
	RNA aptamer	*β*-Catenin	5	Colon cancer	[31]
	RNA aptamer G4	K Ras-derived peptide	139 ± 12	Oncogenic protein	[32]
	RNA aptamer	Amyloid beta-peptide A4 (1-40)	29 to 48	Alzheimer's disease	[33]

	Aptamer BC15 (ssDNA aptamer)	Heterogeneous nuclear ribonucleoprotein A1 (hnRNPA1)	111.0 ± 36.9	Breast cancers and liver cancer	[34]
	DNA aptamer HB5	Her 2 (human epidermal growth factor receptor 2)	18.9	Her 2 positive breast cancer	[35]
DNA	DNA aptamer	0mpC	20	Food borne disease	[36]
	DNA aptamer A and C	TTF1 (member of the NK homeodomain transcription factors)	3.36, and 32.5	Primary lung adenocarcinomas	[37]
	SYL3 DNA aptamer	EpCAM (epithelial cell adhesion molecule)	38 ± 9 67 ± 8	Breast cancer (MDA-MB-231), gastric cancer (Kato III), and solid cancer	[38]
	MUC1 DNA aptamer	MUC1 peptide (Mucin-1)	0.135 to 33.38	Tumor marker in neoplastic cell	[39]

Peptide	Peptide aptamer	RAD 51 (pY315)	na	Leukemia	[40]
	Peptide aptamer A8 and A17	HSP70	na	Cancer	[41]
	Peptide aptamer E6	HPV16 E6 oncoprotein	na	HPV positive tumor cells	[42]
	Peptide aptamer AII-7	ErbB2 (avian erythroblastic leukemia viral oncogene homolog 2)	na	Breast cancer	[43]

na: not available.

**Table 2:** A list of aptamers in clinical pipeline.

Name of aptamer	Company	Target	Indication	Current phase	References
Macugen	Pfizer/Eyetech	VEGF	AMD	Approved	[29, 80]
AS1411	Antisoma	Nucleolin	AML	Phase II	[81]
REG1	Regado	Coagulation factor IXa	ACS	Phase III	[82]
ARC1779	Archemix	vWF	TTP	Phase II	[83]
NU172	ARCA	Thrombin	CABG	Phase II	[84, 85]
E10030	Ophthotech	PDGF	AMD	Phase II	[86]
ARC1905	Ophthotech	C5	AMD	Phase I	[84, 85]
NOX-E36	NOXXON	MCP-1 (CCL2)	Type2DN	Phase II	[87]
NOX-A12	NOXXON	SDF-1 (CXCL12)	Cancer	Phase I	[87]
NOX-H94	NOXXON	Hepcidin	Anemia	Phase I	[88]
BAX499 or ARC19499	Baxter/Archemix	TFPI	Hemophilia	Phase I	[84, 89]

AMD: age-related macular degeneration, AML: acute myeloid leukemia, ACS: acute coronary syndrome, vWF: A1 domain of von willebrand factor, TTP: thrombotic thrombocytopenic purpurea, CABG: coronary artery bypass grafting, DN: diabetic nephropathy, CCL2: chemokine (C-C motif) ligand 2, also known as MCP1, CXCL12: chemokine (C-X-C motif) ligand 12, also known as SDF-1*α*, and TFP1: tissue factor pathway inhibitor.

## Abbreviations

SELEX: Systematic evolution of ligand by exponential enrichment
IVT: * In vitro* transcription
SEB: * Staphylococcal enterotoxin* B
Kd: Dissociation constant
rRNA: Ribosomal ribonucleic acid
V3: Third variable loop of HIV gp120
RT: Reverse transcriptase
HIV-1: Human immunodeficiency virus type 1
PDGF: Platelet derived growth factor.
